# Cultivated Winter-Type *Lunaria annua* L. Seed: Deciphering the Glucosinolate Profile Integrating HPLC, LC-MS and GC-MS Analyses, and Determination of Fatty Acid Composition

**DOI:** 10.3390/molecules29163803

**Published:** 2024-08-10

**Authors:** Gina Rosalinda De Nicola, Sabine Montaut, Kayla Leclair, Joëlle Garrioux, Xavier Guillot, Patrick Rollin

**Affiliations:** 1 Research Centre for Vegetables and Ornamental Crops, Council for Agricultural Research and Economics (CREA), Via dei Fiori 8, 51017 Pescia, Italy; 2 School of Natural Sciences, Laurentian University, 935 Ramsey Lake Road, Sudbury, ON P3E 2C6, Canada; 3 Terres Inovia, 270 Avenue de la Pomme de Pin, BP 90635, Ardon, 45166 Olivet, France; j.garrioux@terresinovia.fr; 4 Laboulet Semences, 80270 Airaines, France; xguillot@laposte.net; 5 Institute of Organic and Analytical Chemistry (ICOA), Université d’Orléans, UMR 7311, BP 6759, F-45067 Orléans, Cedex 2, France; patrick.rollin@univ-orleans.fr

**Keywords:** *Lunaria annua*, glucosinolate, isothiocyanate, nervonic acid, erucic acid

## Abstract

*Lunaria annua* L. (Brassicaceae) is an ornamental plant newly identified in Europe as a promising industrial oilseed crop for its valuable very-long-chain monounsaturated fatty acids (MUFAs), especially erucic acid (EA) and nervonic acid (NA). *L. annua* seeds were obtained from annual winter-type plants selected and cultivated in Northern France. Using a systematic multiple-method approach, we set out to determine the profile and content of glucosinolates (GSLs), which are the relevant chemical tag of Brassicaceae. Intact GSLs were analyzed through a well-established LC-MS method. Identification and quantification were performed by HPLC-PDA of desulfo-GSLs (dGLs) according to the official EU ISO method. Moreover, GSL structures were confirmed by GC-MS analysis of the related isothiocyanates (ITCs). Seven GSLs were identified, directly or indirectly, as follows: 1-methylethyl GSL, (1*S*)-1-methylpropyl GSL, (*Rs*)-5-(methylsulfinyl)pentyl GSL, (*Rs*)-6-(methylsulfinyl)hexyl GSL, (2*S*)-2-hydroxy-4-pentenyl GSL, 2-phenylethyl GSL, and 1-methoxyindol-3-ylmethyl GSL. In other respects, the FA composition of the seed oil was determined. Results revealed cultivated *L. annua* seed to be a source of NA-rich oil, and presscake as a valuable coproduct. This presscake is indeed rich in GSLs (4.3% *w*/*w*), precursors of promising bioactive molecules for agricultural and nutraceutical applications.

## 1. Introduction

*Lunaria annua* L. (Brassicaceae) is a biennial flowering plant originating from western Asia and south-eastern Europe, and it has now been naturalized in many temperate areas. Commonly known as honesty, money plant, or silver penny, it is grown as an ornamental plant for its attractive spring flowers and the decorative value of its seedpods. Growing in semi-shady environments, *L. annua* produces green flat seedpods which reveal a paper-like silvery septum as they mature, very popular as everlasting dry flower arrangements [1]. Molecular studies assigned Lunaria to the tribe Biscutelleae, which now contains the genera Biscutella and Megadenia as well as the latiseptate genera Lunaria and Ricotia [2,3].

Besides its ornamental value, interest in *L. annua* is rapidly growing as a new industrial oilseed crop for its unique oil composition, which makes it very attractive for the booming green-chemical market [4]. *L. annua* oil is rich in very-long-chain monounsaturated fatty acids (MUFAs), comprising mainly 40–45% erucic (C22:1 ω9; (*Z*)-docos-13-enoic acid, EA) and 20–25% nervonic acid (C24:1 ω9; (*Z*)-tetracos-15-enoic acid, NA). Lunaria oil has been recently studied as an interesting feedstock for industrial applications such as a biolubricant and a component in biodiesel production [5]. EA is an important oleochemical product with a wide range of uses in metallurgy, machinery, rubber, chemical industry, and other fields because of its hydrophobicity and water resistance [6]. Uncommon in vegetable sources, NA has been detected only in the seed oil of a few plants [7]. Among those, *L. annua* is one of the biggest potential herbaceous species to be cultivated on a large scale as a suitable crop for NA production dedicated to industrial, cosmetic, and medicinal uses. NA has been identified with critical biological functions in medicine and healthcare for brain development and injury repair. Notably, NA is a promising candidate for intervention on multiple sclerosis and other autoimmune diseases, as shown in a study investigating its effect on inflammation in experimental autoimmune encephalomyelitis (EAE) [8].

**Figure 1:**
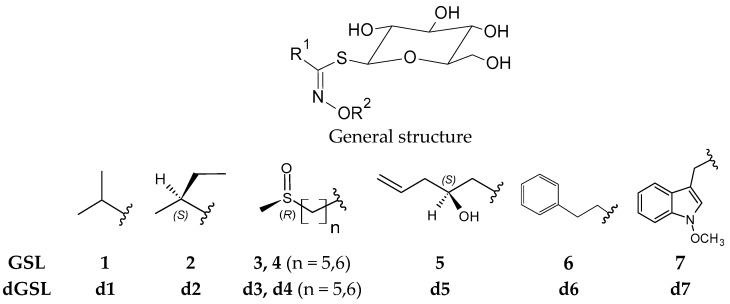
Structures of GSLs (R^2^ = SO_3_^−^) (**1**–**5**) and dGSLs (R^2^ = H) (**d1**–**d7**) identified and quantified in cultivated *Lunaria annua* seed and presscake by LC-MS and HPLC-PDA analysis. Quantification of GSLs was performed by HPLC-PDA analysis of the corresponding dGSLs. Legend: 1-methylethyl GSL (glucoputranjivin) (**1**), (1*S*)-1-methylpropyl GSL (glucocochlearin) (**2**), (*Rs*)-5-(methylsulfinyl)pentyl GSL (glucoalyssin) (**3**), (*Rs*)-6-(methylsulfinyl)hexyl GSL (glucohesperin) (**4**), (2*S*)-2-hydroxy-4-pentenyl GSL (gluconapoleiferin) (**5**), 2-phenylethyl GSL (gluconasturtiin) (**6**), 1-methoxyindol-3-ylmethyl GSL (neoglucobrassicin) (**7**), d-glucoputranjivin (**d1**), d-glucocochlearin (**d2**), d-glucoalyssin (**d3**), d-glucohesperin (**d4**), d-gluconapoleiferin (**d5**), 2-phenylethyl dGSL (d-gluconasturtiin) (**d6**), and 1-methoxyindol-3-ylmethyl dGSL (d-neoglucobrassicin) (**d7**).

Glucosinolates (GSLs) are specialized metabolites whose structure consists of an *S*-*β*-D-glucopyrano unit anomerically connected to an *O*-sulfated (*Z*)-thiohydroximate function (Figure 1). To date, there are 88 satisfactorily characterized GSLs and an additional 49 partially characterized GSLs, so the total number of unique GSL structures falls between 88 and 137 [9]. GSLs are widely distributed among plants belonging to the order Brassicales including the Brassicaceae family. Like all members of the Brassicaceae family, *L. annua* contains GSLs and the enzyme myrosinase (EC 3.2.1.147), which catalyzes the hydrolysis of GSLs, affording a diversity of degradation products such as isothiocyanates (ITCs), recognized as functional substances with wide applications within pharmaceutical, nutraceutical, cosmetic, and agricultural industries [9,10]. Notwithstanding its high potentiality as a multipurpose crop, cultivated *L. annua* has suffered from low oil content of about 25–35% and unstable yield varying widely between 800 and 2000 kg ha^−1^ [11,12,13]. Moreover, the biennial nature of *L. annua* is a main constraint for its cultivation which has so far proved uneconomic for commercial production. Significant research has been conducted since 2010 by Laboulet Semences, a seed company based in France, performing breeding and cultivation trials to develop an annual winter-type variety aiming for a viable supply chain of *L. annua* oil rich in EA and NA. Notably, the presence of GSLs in the presscake obtained as a coproduct of oil production may increase the portfolio of biobased-derived products and, consequently, the crop economy. However, only a limited number of studies have described *L. annua* GSLs and the potentiality of its degradation products so far [14,15,16]. Recently, GSLs in *L. annua* seeds were analyzed via their desulfo-GSLs (dGSLs) using the UHPLC-DAD-MS/MS technique and by their volatile breakdown products such as ITCs, using GC-MS. In that study, GSL breakdown products were obtained by hydrodistillation in a Clevenger-type apparatus and dichloromethane extraction after 24 h myrosinase hydrolysis, as well as by microwave-assisted distillation and microwave hydrodiffusion and gravity. Isopropyl-, *sec*-butyl-, 5-(methylsulfinyl)pentyl-, 6-(methylsulfinyl)hexyl-, 5-(methylsulfanyl)pentyl-, 6-(methylsulfanyl)hexyl-, and benzyl GSLs were identified. Additionally, pent-4-enyl- and hex-5-enyl ITCs were detected in the volatile extracts [16].

In this study, we investigated an annual winter-type *L. annua* selected by the Laboulet Semences company and cultivated in Northern France. As a part of our continued interest in the chemistry of Brassicales, the first objective of this investigation was to determine the qualitative and quantitative GSL profile in the whole seed and in the presscake using a multiple-method approach. First, a systematic investigation using a well-established LC-MS method of identification of intact GSLs of seeds was used. This is a direct method that allows us to obtain a quick GSL profile without any degradation products. Next, the identification and quantification were conducted by HPLC-PDA analysis of dGSLs, according to the ISO 9167:2019 method [17]. This is the standard method of GSL analysis that is widely used, allowing us to compare our results with other published data. Moreover, GSL structures were confirmed indirectly by GC-MS analysis of isothiocyanates (ITCs). This is also an indirect method of GSL identification. The use of these three complementary methods provides a comprehensive analysis of the GSL composition. Finally, the second objective of this study was to assess the fatty acid (FA) composition of *L. annua* oil by GC-FID according to the ISO 12966-4 [18] method, which is a standard method of FA analysis.

## 2. Results and Discussion

### 2.1. Whole Seed Quality

Cultivated winter-type *L. annua* seed harvested in Amiens (France) was evaluated for GSL profile and content as well as for ITC identification of GSL degradation. The presscake obtained as a coproduct of oil pressure was also analyzed for GSLs. Moreover, FA composition was established for oilseed quality through a rapid method.

#### 2.1.1. Intact Glucosinolate LC-MS Analysis

**Figure 2:**
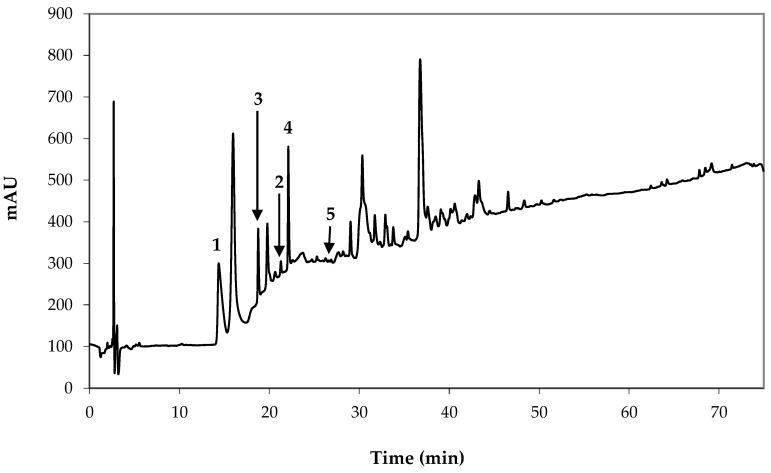
HLPC chromatogram of the ethanolic seed extract of *Lunaria annua* L. Detection at 220 nm. Legend: 1-methylethyl GSL (glucoputranjivin) (**1**), (1*S*)-1-methylpropyl GSL (glucocochlearin) (hypothesis) (**2**), (*Rs*)-5-(methylsulfinyl)pentyl GSL (glucoalyssin) (**3**), (*Rs*)-6-(methylsulfinyl)hexyl GSL (glucohesperin) (**4**), (2*S*)-2-hydroxy-4-pentenyl GSL (gluconapoleiferin) (hypothesis) (**5**).

The profile of intact GSLs was determined using an HPLC-ESI-MS method (Figure 1 and Figure 2). Five known GSLs were identified as follows: two with a branched alkyl short chain: 1-methylethyl GSL (glucoputranjivin, **1**) and (*S*)-1-methylpropyl GSL (glucocochlearin, **2**) originating from the Val and Ile biosynthetic pathways, respectively; two with a thiofunctionalized chain: (*Rs*)-5-(methylsulfinyl)pentyl GSL (glucoalyssin, **3**) and (*Rs*)-6-(methylsulfinyl)hexyl GSL (glucohesperin, **4**); and one with an alkenyl chain: (2*S*)-2-hydroxy-4-pentenyl GSL (gluconapoleiferin, **5**); the last three all originated from the Met biosynthetic pathway. GSLs **1**, **3**, and **5** were identified from their t_R_, UV, and mass spectra as compared to those of previously isolated GSLs in our laboratory [19,20,21]. GSLs **2** and **5** were hypothesized on the basis of our LC-MS data and comparison of the similarities of spectroscopic data with GSLs previously isolated in our laboratory. ESI-MS values for **1**, **3,** and **4** showed agreement with previously published analyses of *L. annua* seed [14,22].

#### 2.1.2. Desulfoglucosinolate HPLC Profiling and Quantitative Analysis by Laboratory A (CREA Research Centre for Vegetable and Ornamental Crops, Pescia, Italy)

**Figure 3:**
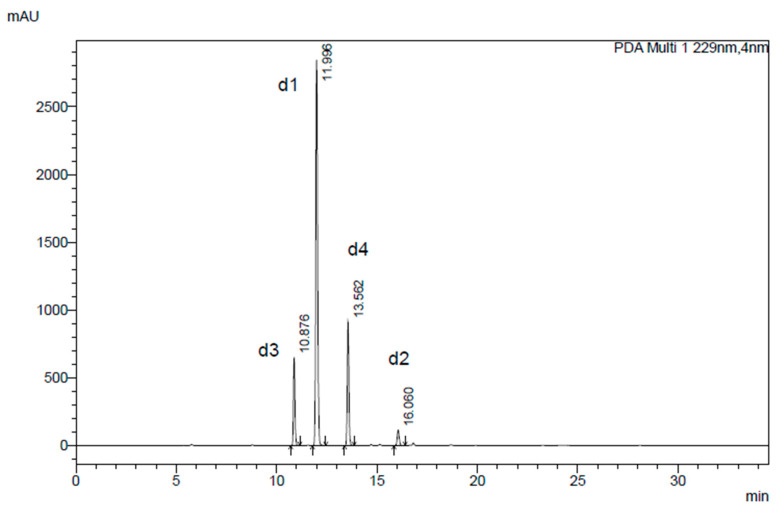
HLPC-PDA chromatogram of dGSLs obtained from the ethanolic seed extract of *Lunaria annua* L (Laboratory A). Detection at 229 nm. Legend: 1-methylethyl dGSL (d-glucoputranjivin) (**d1**), (1*S*)-1-methylpropyl dGSL (d-glucocochlearin) (**d2**), (*Rs*)-5-(methylsulfinyl)pentyl dGSL (d-glucoalyssin) (**d3**), (*Rs*)-6-(methylsulfinyl)hexyl dGSL (d-glucohesperin) (**d4**).

Seed ethanolic extracts were analyzed by HPLC-PDA to detect the dGSLs resulting from desulfation of GSLs according to the EU standard procedure ISO 9167:2019 [17]. The identity of each dGSL was determined by the comparison of the t_R_ and UV spectra with those of dGSL standards. Four peaks were detected and identified as two branched alkyl short-chain dGSLs, d-glucoputranjivin **d1** and d-glucocochlearin **d2**, and two methylsulfinylalkyl dGSLs, d-glucoalyssin **d3** and d-glucohesperin **d4** (Figure 1 and Figure 3). The chromatogram showed three major peaks **d1**, **d2**, and **d3** in agreement with previous reports on GSL analysis in *L. annua* seed as their desulfo-counterparts by using LC-API-MS and UHPLC-MS/MS [16,23]. The GSL content was quantified using a calibration curve of pure d-sinigrin (2-propenyl dGSL) solution (range from 0.06 to 1.93 mM) and the relative proportionality factors (RPFs) reported in the literature for **d3** and **d1** [24,25]. Compound **d4** was assumed to have the same RPF as **d3**, whereas an arbitrary RPF = 1 value was taken for **d2**. The quantitative GSL profile established for *L. annua* seed is reported in Table 1. The total GSL content in cultivated *L. annua* seed is rather high, with a value of 2.7% (*w*/*w*) calculated for whole non-defatted seed. The most abundant GSL is **1,** representing 65% of total GSLs, followed by **4** and **3,** accounting for 33% of total GSLs, and **2** accounting for a minor part. In a previous study, the same three major GSLs were identified in a commercially available seed of *L. annua* analyzed using an optimized ion-pairing LC-MS method. Moreover, our quantification is in line with the range of concentration given in the same report [14].

#### 2.1.3. Isothiocyanate GC-MS Analysis

**Figure 4:**
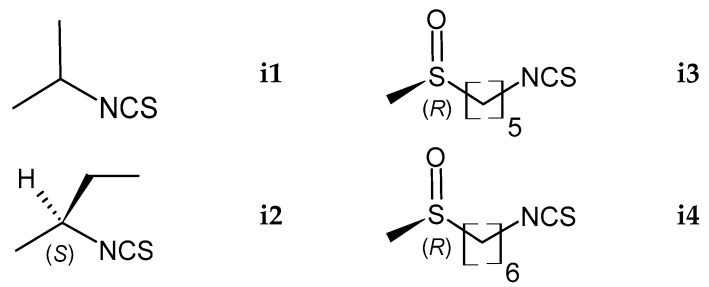
Structures of ITCs issued from enzymatic hydrolysis of GSLs and identified by GC-MS analysis. Legend: 1-methylethyl ITC (putranjivin) (**i1**), (1*S*)-1-methylpropyl ITC (**i2**), (*Rs*)-5-(methylsulfinyl)pentyl ITC (*R*-alyssin) (**i3**), (*Rs*)-6-(methylsulfinyl)hexyl ITC (*R*-hesperin) (**i4**).

ITCs were produced by myrosinase hydrolysis of the crude seed hydroalcoholic extract at neutral pH. GC-MS analysis was performed using two different injector port temperatures. In a typical experiment with the injector port temperature set at 250 °C, six ITCs were detected and univocally identified as reported in Table 2 and Figure 4.

Isopropyl ITC (putranjivin, **i1**, peak 1), (1*S*)-1-methylpropyl ITC (**i2**, peak 2), 4-pentenyl ITC (brassicanapin, peak 3), 5-hexenyl ITC (peak 4), (*Rs*)-5-(methylsulfinyl)pentyl ITC (alyssin, **i3**, peak 5), and (*Rs*)-6-(methylsulfinyl)hexyl ITC (hesperin, **i4**, peak 6) were identified from their ion fragmentation patterns and t*_R_* compared to available mass spectra and literature data [26,27,28,29,30,31]. The GSL degradation products **i1**, **i2**, **i3**, and **i4** resulting from myrosinase hydrolysis confirmed the presence of GSLs identified by HPLC-PDA analysis. Additional identification of two alkenyl ITCs, namely 4-pentenyl ITC and 5-hexenyl ITC, is the first unambiguous demonstration that such compounds are artifacts originating in the spectrometer injection port. By promoting the degradation of terminal sulfoxides, higher temperatures caused the conversion of alyssin and hesperin into 4-pentenyl ITC and 5-hexenyl ITC, respectively. Thus, when lowering the injection temperature from 250 °C to 180 °C, the latter compounds were not detected (Table 2). In a previous paper, we speculated the same thermal degradation of alyssin to 4-pentenyl ITC when studying the GL composition of *Aurinia leucadea* (Guss.) C. Koch and *A. sinuata* (L.) Griseb. [21].

Gas chromatography (GC) is certainly a convenient technique for ITC analysis; however, several ITCs are thermally unstable. Chiang et al. first identified 3-butenyl ITC as a primary thermal degradation product of (*Rs*)-4-(methylsulfinyl)butyl ITC (sulforaphane) in the GC injection port. Consequently, those authors developed a GC-MS method wherein thermal degradation of sulforaphane was significantly reduced, due to modifications of GC-MS operating parameters [32].

**Figure 5:**
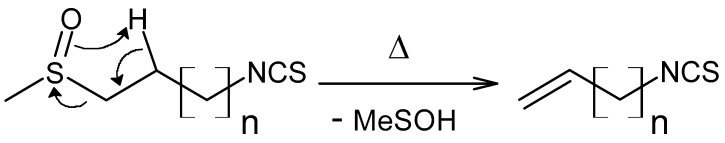
Proposed mechanistic scheme for the thermal decomposition of (*Rs*)-5-(methylsulfinyl)pentyl ITC (*R*-alyssin) (**i3**, n = 5) and (*Rs*)-6-(methylsulfinyl)hexyl ITC (*R*-hesperin) (**i4**, n = 6) to the corresponding 4-pentenyl ITC (brassicanapin, n = 5) and 5-hexenyl ITC (n = 6) detected by GC-MS in the experiment with the injector port set at 250 °C.

Our results underline that the ITCs detected by GC-MS analysis do not necessarily match those resulting from the hydrolysis of GLs, depending on their side chain. Indeed, alkyl GSLs bearing a terminal sulfoxide moiety on their side chain can undergo thermally induced β-elimination of methanesulfenic acid as suggested by Cedrowski et al. (2021) [33] for the conversion of sulforaphane into 3-butenyl ITC. Figure 5 shows a proposed path for the thermal decomposition of alyssin **i3** and hesperin **i4** to afford the alkenyl ITCs detected by GC-MS.

#### 2.1.4. Presscake Glucosinolate Content

*L. annua* seeds were triturated via a cold-pressing procedure using a laboratory Komet oil mill with a 21% oil extraction yield. Cold press extraction is widely used to produce solvent-free high-quality vegetable oils at low temperatures. This environmental friendly mechanical extraction offers several advantages including simple use, short duration of the process, and low cost. Furthermore, it is suitable for small seed quantities, and the low temperature ensures the quality of the presscake as a byproduct with preserved features such as phytochemical profile and content. The quality of the presscake obtained from lunaria seed developed by Laboulet Semences was evaluated by external Laboratory B (Terres Inovia, Ardon, France) through dGSL analysis. Table 3 summarizes the results obtained by Terres Inovia for the whole seed and the presscake obtained after oil cold pressure extraction. The results of dGSL analysis were pretty consistent with those obtained by Laboratory A (CREA Research Centre for Vegetable and Ornamental Crops, Pescia, Italy) for the whole seed, except for the detection of additional minor d-gluconasturtiin **d6**.

*L. annua* presscake has proven to be a valuable coproduct of oil extraction. Indeed, with a total GSL content of 112 µmol g^−1^, it can be labeled as a high-GSL feedstock suitable for exploitation as a green material, opening opportunities for improved economic and environmental sustainability of *L. annua* as an industrial crop. Looking at this cultivation according to a biorefinery approach, the plant (first-generation biorefinery) produces a level of total GSL accumulation in seeds of 2.7% (*w*/*w*) (Laboratory A 2.7%, Laboratory B 2.6%). The cold-pressing technique represents a simple way to obtain the presscake as a second-generation product with an enriched GSL content up to a total of 4.3% (*w*/*w*). Consistently with the whole seed, *L. annua* presscake is particularly rich in glucoputranjivin **1** (71% of total GSLs), glucohesperin **4** (13% of total GSLs), and glucoalyssin **3** (12% of total GLs), three important GSLs studied for their properties and the biological activity of their corresponding ITCs. Laboratory B detected **d2**, confirming gluconapoleiferin **2** being present in *L. annua* seed, as well as additional minor **d6** and **d7**.

#### 2.1.5. *Lunaria annua* Presscake Prospects

Within the context of an accelerating biobased economy, a growing effort is being made to develop substitutes for petroleum-based products from agricultural renewable feedstock. Our findings indicate *L. annua* as a significant source of sustainable raw materials, enabling the manufacture of biobased options including biolubricants, products for agriculture, and nutraceuticals.

In a previous study, a solvent-defatted *L. annua* seed meal was investigated for potential bioherbicidal activity amongst a total of fifteen seed meals from different GSL-containing plants. The efficacy was evaluated in terms of seed germination inhibition on two target weed species, namely wheat (*Triticum aestivum* L. var. Cardinal) and sicklepod (*Senna obtusifolia* L. H. S. Irwin & Barneby). Seed meals from *L. annua*, Indian mustard (*Brassica juncea*), and field pennycress (*Thlaspi arvense*) proved the most phytotoxic, completely suppressing germination at all concentrations tested. Such results suggested that seed meals containing short-chain aliphatic GSL such as **1**, sinigrin (2-propenyl GSL), and gluconapin (3-butenyl GSL) were among the most inhibitory, through producing volatile **i1**, allyl ITC and 3-butenyl ITC, respectively [15].

Rich sources of glucoputranjivin **1** are quite limited as **1**-containing species are not very widespread amongst Brassicaceae, with the highest levels having been reported only in seeds of *L. annua* and *Sisymbrium officinalis* (L.) Scop. (hedge mustard), a wild plant known as the singers’ plant for its traditional use in treating aphonia and vocal disability. Both intact bioprecursor **1** and the related ITC **i1** isolated from fresh hedge mustard were tested on the transient receptor potential ankyrin 1 (TRPA1) channel, which is involved in the somatosensory perception of pungency as well as in the nociception pathway of inflammatory pain. In contrast to inactive **1**, compound **i1** proved to be a potent agonist of TRPA1, with an EC_50_ in the range of the high-potency natural agonists identified so far for this somatosensory channel [34]. The same authors identified **1** as a selective in vitro agonist of the T2R16 receptor, one of the T2R platform of bitter taste receptors [35]. Di Sotto (2012) [36] previously reported the antimutagenic activity of **1** for the first time using a bacterial reverse mutation assay (Ames test) on different *Escherichia coli* strains. GSL **1** is currently under evaluation for the potential neuroprotective properties in brain cell models (personal communication).

*L. annua* presscake is a convenient source for the preparation of GSL-enriched extracts as well as an optimal starting material suitable for the isolation of pure **1**. Indeed, the presscake contains a high percentage (2.9% *w*/*w*) of **1**, which is also the most abundant among a limited number of GSLs. Those data fulfill the starting conditions to make it a remarkable candidate for the efficient purification of **1** through a simple gram-scale preparative procedure, as previously described [37]. Furthermore, **1** can easily be further transformed directly into the corresponding ITC **1c** in a biphasic system without the requirement for any chromatographic step, as already reported for the production of *R*-sulforaphane from Tuscan black kale defatted seed meal [37].

Thiofunctionalized GSLs **3** and **4** are homologs of glucoraphanin (*R*_S_)-4-(methylsulfinyl)butyl GSL, the precursor of popular *R*-sulforaphane, one of the most studied dietary ITCs which is emerging as a therapeutic option for decreasing the risk of development of several chronic diseases. The beneficial effects of *R*-sulforaphane are well known and widely reported [38]. Structurally close to *R*-sulforaphane, **i3** and **i4** were also identified in wasabi and horseradish. Those homologous methylsulfinylalkyl ITCs have shown diverse biological activities including antibacterial, fungicidal, CYP-inhibitory, and antiproliferative properties, and they are attracting great attention as new possible candidates for controlling cancer cell progression and metastasis [39].

The GSL composition of the presscake of other cultivated oleaginous seeds of the Brassicaceae family has been studied. For comparison purposes, we report here some recent research findings. Rapeseed presscakes containing 30–40% high-quality protein have been the subject of considerable attention as an alternative source of protein for various food and feed applications. Several GSLs in cold-pressed rapeseed cake were identified, with the main compounds being gluconapin (0.2% *w*/*w*) and progoitrin (0.1% *w*/*w*) [40]. Oilseed presscakes are critical crop commodities for many industries. Interest in presscake extracts is also increasing for the production of high-added-value molecules and products from agricultural materials. Major sinigrin (5.5% *w*/*w*) and minor gluconapin (0.7% *w*/*w*) were found in mustard seed (*Brassica juncea*) presscake. By using an innovative pretreatment (high-voltage electrical discharges) and green solvents, an eco-friendly process was established, to simultaneously recover a GSL-rich extract, and a detoxified meal rich in proteins suitable for animal feeding. The obtained mustard presscake extract rich in sinigrin may be used as a sustainable source of degradation products with biopesticide action to replace agrochemicals [41]. False flax (*Camelina sativa* L.), commonly known as camelina, represents one ancient oil plant that has gathered renewed interest. Camelina, belonging to the Brassicaceae family alongside rapeseed and mustard, shares GSLs typical of these plants. However, the GSL profile of camelina is unique, consisting solely of three aliphatic compounds with long chains: glucoarabin ((*R*_S_)-9-(methylsulfinyl)nonyl GSL, GSL9), glucocamelinin ((*R*_S_)-10-(methylsulfinyl)decyl GSL, GSL10), and (*R*_S_)-11-(methylsulfinyl)undecyl GSL (GSL11). As homologs of renowned glucoraphanin, camelina GSLs have great potential to be further investigated for their antioxidative effects [42]. Recently, camelina presscake obtained by a commercially available seed was examined for GSL content displaying GSL10 followed by minor GSL9 and GSL11 being present in present in 4.2%, 0.9, and 0.7% (*w*/*w*), respectively [43].

#### 2.1.6. *Lunaria annua* Seed Fatty Acid Composition

The investigated winter-type *L. annua* seed was characterized by a high oil content value of 36.8%. In particular, this selection contained exceptionally high levels of NA, which is uncommon in vegetable sources [7]. Over 90% of the FAs in *L. annua* oil were monounsaturated with major EA, oleic acid (OA), and NA present in 44.6%, 24.2%, and 21.8%, respectively (Table 4).

Total MUFAs, mainly represented by EA, OA, and NA, accounted for almost 90% of total FAs with minor polyunsaturated FAs (7%) and saturated FAs (2%). A previous report evaluated nineteen species from eleven different genera of the family Brassicaceae for utility as edible oil as well as renewable industrial or fuel oils. The total MUFAs, represented by the sum of the content of oleic (C18:1), gondoic (C20:1), and erucic (C22:1) acids in the seed oils, ranged from 38.41% in *Capsella bursa pastoris* to 79.89% in *Crambe abyssinica* [44]. Similarly, the EA percentage of 45% fell on the high side of the 28–56% range reported for 11 cultivated species of non-food Brassicaceae seeds, namely *Barbarea verna*, *Brassica carinata*, *Brassica juncea*, *Brassica napus*, *Brassica nigra*, *Brassica rapa*, *Cheirantus cheiri*, *Crambe abyssinica*, *Eruca sativa* spp. *oleifera*, *Iberis amara*, and *Sinapis alba* [45]. The exclusive presence of 23% of NA is a remarkable added value of the investigated *L. annua* crude oil to be considered as a feedstock for the pharmaceutical industry, as NA is recognized to support the normal function of myelin in brain and nerve tissue. However, unmodified *L. annua* oil is not viable in supplementation, because it does not meet the nutraceutical and pharmaceutical NA oil requirements (high NA content but very low EA (<5%) content), as diets rich in EA may have toxic effects on the heart, impairing myocardial conductance and increasing blood cholesterol. Current research programs aim to transfer strategic *L. annua* genes to other well-established Brassica oilseed crops for engineering high-NA oil production. Anyway, the use of NA in chemical applications is promising with high potential for the synthesis of nylon 15 and the production of polyesters [7].

The composition of the FA of oil obtained by the cold pressing of other cultivated oilseed plants of the Brassicaceae family has been studied. For comparison purposes, we cite here some references. In a previous investigation, rapeseed oil was shown to contain OA (C18:1) as a major FA (63.1%), followed by linoleic acid (C18:2) (19.49%) and linolenic (C18:3) [46]. Furthermore, cold-pressed camelina oil is known to contain, in descending order, linolenic acid (34%), linoleic acid (19.4%), oleic acid (18.0%), gondoic acid (14.8%), and palmitic acid (5.2%) [43].

## 3. Materials and Methods

### 3.1. Chemicals

All solvents were ACS grade and used as such. Formic acid was purchased from BDH (Toronto, ON, Canada). HPLC-grade MeOH and Et_3_N (reagent grade) were purchased from Fisher Scientific (Whitby, ON, Canada). HPLC-grade H_2_O was generated in a laboratory through a Nanopure Diamond Ultrapure water system provided by Barnstead (Dubuque, IA, USA) for the LC-MS analysis of intact GSLs. DEAE Sephadex A-25 resin was purchased from GE Healthcare (Freiburg, Germany). Analytical-standard sinigrin was obtained from Supelco. Myrosinase and sulfatase enzymes were obtained from Sigma-Aldrich Chemie (Steinheim, Germany). Water, acetonitrile, and dichloromethane were HPLC grade from Sigma-Aldrich Chemie (Steinheim, Germany) for HPLC-PDA analysis of dGSLs and for GC-MS analysis of ITCs.

### 3.2. Plant Material

*L. annua* seeds were obtained from cultivated winter annual-type plants selected by the company Laboulet Semences (Airaines, France). Seeds were grown in Amiens (France) after a summer sowing using improved agronomic conditions (information available on request). The voucher specimen (LUNARIA 2-6 Amiens (80)) was deposited at the Institute of Organic and Analytical Chemistry (ICOA) University of Orléans, Orléans, France. *L. annua* seeds were extracted by a cold-pressing procedure using a laboratory Komet oil mill. The resulting presscake was analyzed by Laboratory B Terres Inovia (Ardon, France) for dGSL identification and quantification (see Section 3.3.5).

### 3.3. Whole Seed Analyses

#### 3.3.1. Moisture and Oil Content

Lunaria seed was subjected to determination of its moisture and oil content by the following methods:

(i) Moisture content was determined by Laboulet Semences (Airaines, France) by oven-drying the seeds (10 g) at 101–105 °C for 24 h and calculated as the difference between the seed weight before and after treatment, according to the French standard NF V03-909 method [47]. Determinations were performed in duplicate.

(ii) Oil content was established by Terres Inovia (Ardon, France) according to the standard NF V03-908 simplified method [48]. About 1 g of seed was ground in hexane (70 °C) using a Dangoumau-type mill with a steel ball. Oil was then extracted for 3 h with hexane by the Soxhlet method. The seed oil content was expressed as a percentage of the whole seed weight. Determinations were performed in duplicate.

#### 3.3.2. LC-MS Analysis of Intact Glucosinolates

*Lunaria annua* L. seeds (507.7 mg) were frozen in liquid nitrogen, ground in a mortar, and immediately extracted two times with boiling EtOH-H_2_O (5 mL, 7:3, *v*/*v*) for 5 min. The ethanolic solution was filtered and concentrated to dryness (78.0 mg). LC-MS analysis was performed by injecting a 5 μL aliquot of the 15 mg mL^−1^ crude extract into an Agilent Technologies HP 1100 (New Castle, DE, USA) high-performance liquid chromatograph equipped with a quaternary pump, automatic injector, diode-array detector (wavelength range 190–600 nm), degasser, and a Hypersil ODS column (5 μm, 4.6 × 200 mm). The two mobile phase solvents (MeOH and H_2_O) were prepared with 0.15% Et_3_N and 0.18% HCO_2_H, added as ion-pairing reagents. Both solutions were filtered through 0.45 μm nylon membranes. The initial mobile phase was 100% HPLC-grade H_2_O. At 10 min, the mobile phase was switched to a linear gradient of 100% H_2_O to 100% MeOH over 60 min. After each run, the initial mobile phase conditions were set, and the system was allowed to equilibrate. The flow rate was kept at a constant 1 mL min^−1^. The column temperature was held at 32 °C [49]. The HPLC was interfaced to an Agilent model 6120 mass spectrometer (Toronto, ON, Canada) with a Chemstation LC-MSD B.03.01 data system. The electrospray interface was a standard ED source operating with a capillary voltage of 4 kV and a temperature of 350 °C. Spectra were obtained with a fragmentation voltage of 200 eV. The system was operated in negative and positive ion electrospray modes. Nitrogen was used as drying gas at a flow rate of 12 L min^−1^ (60 psig). The mass spectrometer was programmed to perform full scans between *m*/*z* 100 and 1500 amu.

#### 3.3.3. HPLC-PDA Analysis and Quantification of Desulfoglucosinolates by Laboratory A (CREA Research Centre for Vegetable and Ornamental Crops, Pescia, Italy)

GSLs were analyzed according to the EU standard procedure ISO 9167:2019 [17] with some modifications. Ripe *L. annua* seeds were reduced to a fine powder. Samples of seed (*ca*. 600 mg) were extracted for 5 min at 80 °C twice with EtOH-H_2_O (5 mL, 7:3 *v*/*v*), using a VDI 25 homogenizer (VWR, Darmstadt, Germany) and then centrifuged with a 5810R centrifuge (Eppendorf AG, Hamburg, Germany) at 4000× *g* rpm for 10 min at 10 °C. Supernatants were combined, and each extract (1 mL) was loaded onto a mini-column filled with DEAE-Sephadex A-25 anion-exchange resin (0.6 mL, GE Healthcare) conditioned with 25 mM sodium acetate buffer (pH 5.6). After washing with buffer (3 mL), purified sulfatase (200 µL) was loaded onto the mini-column and left for overnight reaction. Extraction and desulfation were performed in duplicate. DGSLs were then eluted with ultra-pure H_2_O (3 mL) and analyzed on an HPLC-PDA LC-40 model (Shimadzu, Kyoto, Japan) system equipped with an Avantor ACE 5 C18 column (250 × 4.6 mm, 4 μm particle size) (VWR, Darmstadt, Germany), thermostated at 30 °C, and having a PDA detector. The chromatography was performed at a flow rate of 1 mL min^−1^, eluting with a gradient of H_2_O (A) and CH_3_CN (B) with the following program: 1 min 1% B; 33 min linear gradient up to 33% B; hold at 33% B for 7 min; 3 min linear gradient down to 1% B. DGSLs were detected by absorbance monitoring at 229 nm. Identification of the peaks was performed based on retention times and UV spectra of standards available in our library. The GSL amount was quantified by using a calibration curve of pure desulfo-sinigrin solution (range from 0.06 to 1.93 mM) and the RPF of each individual dGSL [24,25]. RPF values for quantification were as follows: 1.07 for d-glucoalyssin **d3** and d-glucohesperin **d4**, 1.0 for d-glucoputranjivin **d1** and d-glucocochlearin **d2**.

#### 3.3.4. Isothiocyanate Production through Myrosinase-Catalyzed Hydrolysis of Glucosinolates

Isothiocyanates were produced through a well-established procedure used at our GSL laboratory [50]. Seed ethanolic crude extract (1 mL) was concentrated to dryness with a rotary evaporator and the residue dissolved in 0.1 M potassium phosphate buffer, pH 7 (1 mL). ITCs were produced by enzymatic hydrolysis of GSLs via addition of myrosinase (β-thioglucoside glucohydrolase EC 3.2.1.147, 100 µL, 30 U mL^−1^) to the buffered solution. After 15 min at 37 °C, the resulting ITCs were extracted with CH_2_Cl_2_ (1 mL) and analyzed by GC-MS after drying over K_2_SO_4_, as already reported [50].

#### 3.3.5. GC-MS Analysis of Isothiocyanates

GC-MS analyses were carried out using a Bruker Scion SQ Premium (Bruker Daltonics, Macerata, Italy) equipped with a 30 m × 0.25 mm capillary column HP-5ms. The flow rate of the carrier gas (He) was 1 mL min^−1^. Temperature programming was from 50 °C (hold 2 min) to 260 °C at a rate of 8 °C min^−1^ (hold 1 min). The analysis was run under two different injector port temperatures of 250 °C and 180 °C to avoid thermal degradation of hesperin and alyssin [39]. The temperature of the detector was set at 280 °C. All MS analyses were performed in the electron impact (EI+) mode at 70 eV, the mass range being from *m*/*z* 40 to 650 and the chromatogram acquired in total ion current (TIC). The identification of ITCs was assigned by matching the recorded mass spectra with available standards and comparing them with the literature.

#### 3.3.6. HPLC-DAD Analysis and Quantification of Desulfoglucosinolates in Whole Seed and Presscake by Laboratory B (Terres Inovia, Ardon, France)

An external laboratory (Terres Inovia, Ardon, France) was in charge of providing GSL determinations. HPLC-DAD analysis of the corresponding dGSLs was carried out on the whole seed and the presscake according to the official ISO 9167:2019 [17].

#### 3.3.7. Fatty Acid Extraction and Determination

The FA profile was established by Terres Inovia (Ardon, France) after extraction of ground seed with hexane and transmethylation of triglycerides in KOH methanolic solution according to the ISO 12966-2 method [51]. The resulting fatty methyl esters were further analyzed by GC-FID according to ISO 12966-4 [18]. The content of each FA was expressed as a percentage of total FA content.

## 4. Conclusions

This report presents analytical results for the evaluation of a cultivated annual winter-type *L. annua* seed and its presscake, an interesting coproduct of oil extraction. In the present study, we have directly or indirectly identified GSLs **1**–**7** in *L. annua* seed and presscake. In addition, we determined that EA was the major FA of the seed oil followed by OA and NA. *L. annua* oil constitutes an attractive feedstock for biolubricant production and for the pharmaceutical and cosmetic sectors because of its unusual FA profile with a high NA content. Moreover, the remarkable profile and content of the GSLs found in the presscake suggest promising high-value applications in the nutraceutical industry for health improvement and in agriculture for a sustainable weed management strategy. *L. annua* hence appears as a multifunctional crop with major uses as a source of oil, NA, and GSLs; it should be considered for further investigation as a raw material for the production of biopesticides and nutraceuticals.

## Figures and Tables

**Table 1:** Glucosinolate (GSL) mass spectrometry data and content in whole seed of cultivated winter annual *Lunaria annua* (determined by Laboratory A).

Glucosinolate (GSL)				Whole Seed Content			
#	Class	Semisystematic Name (*Trivial Name*)	[M − H] ^1^ *m*/*z* (%)	µmol g^−1^	mg g^−1^	% on Total GSLs	% (*w*/*w*)
**1**	Val	1-Methylethyl GSL (*glucoputranjivin*)	359.8 (100)	44.58 ± 1.10 ^2,3^	16.11 ± 0.40	64.75	1.61
**2**	Ile	(1*S*)-1-Methylpropyl GSL (*glucocochlearin*)	374.0 (100)	1.76 ± 0.05	0.66 ± 0.02	2.56	0.07
**3**	Met	(*Rs*)-5-(Methylsulfinyl)pentyl GSL (*glucoalyssin*)	449.8 (100)	8.87 ± 0.20	4.00 ± 0.09	12.88	0.40
**4**	Met	(*Rs*)-6-(Methylsulfinyl)hexyl GSL (*glucohesperin*)	464.0 (100)	13.64 ± 0.74	6.35 ± 0.35	19.81	0.63
**5**	Met	(2*S*)-2-Hydroxy-4-pentenyl GSL (*gluconapoleiferin*)	402.0 (100)	-	-	-	-
Total				68.85 ± 2.09	27.12 ± 0.86	100	2.71

^1^ ESI^−^-MS data; ^2^ duplicate; ^3^ standard deviation.

**Table 2:** Glucosinolate hydrolysis products identified by GC-MS in cultivated winter annual *Lunaria annua* seeds.

Isothiocyanates (ITCs)				
Peak #	Semisystematic Name (*Trivial Name*)	Precursor Compound	Injector Port T (°C) ^a^	MS Spectral Data (*m*/*z*) ^b^
1	1-Methylethyl ITC (*putranjivin*)	1-Methylethyl GSL (*glucoputranjivin*)	180, 250	**101**, 86, 72, 60, 59, 58, 43, 42, 41, 39 Lit. ^c,d,e^
2	(1*S*)-1-Methylpropyl ITC	(1*S*)-1-Methylpropyl GSL (*glucocochlearin*)	180, 250	115, 86, 72, 57, 56, **41**, 39 Lit. ^c,d,e^
3	4-Pentenyl ITC (*brassicanapin*)	(*Rs*)-5-(Methylsulfinyl)pentyl ITC (*R*-*alyssin*)	250	127, 126, 112, 99, 85, 72, 70, 67, 60, 59, 58, 55, 53, **41**, 39 Lit. ^c,d,f^
4	5-Hexenyl ITC	(*Rs*)-6-(Methylsulfinyl)hexyl ITC (*R*-*hesperin*)	250	141, 140, 126, 113, 108, 97, 84,72, 67, 55, 54, **41**, 39 Lit. ^d^
5	(*Rs*)-5-(Methylsulfinyl)pentyl ITC (*R*-*alyssin*)	(*Rs*)-5-(Methylsulfinyl)pentyl GSL (*glucoalyssin*)	180, 250	174, 128, **72**, 69, **41**, 39 Lit. ^g^
6	(*Rs*)-6-(Methylsulfinyl)hexyl ITC (*R*-*hesperin*) ^b^	(*Rs*)-6-(Methylsulfinyl)hexyl GSL (*glucohesperin*)	180, 250	188, 142, 126, 72, **55**, 41, 39 Lit. ^g,h^

^a^ This column indicates in which experiment the compounds were detected in the GC-MS analysis with injection port temperature set at 180 °C or 250 °C. ^b^ base ion in bold. Literature: ^c^ [26]; ^d^ [27]; ^e^ [28]; ^f^ [29]; ^g^ [30]; ^h^ [31].

**Table 3:** GSL profile and content in whole seed and presscake of cultivated winter-type *Lunaria annua* determined by Laboratory B (Terres Inovia, Ardon, France) by HPLC-DAD of dGSLs. Humidity and oil content (%) are given for the whole seed.

	Humidity (%)	Oil (%)	Glucosinolate Content															
			(µmol g^−1^)								% (*w*/*w*)							
			d1	d2	d3	d4	d5	d6	d7	Total	1	2	3	4	5	6	7	Total
Whole seed	8.7	36.8	47.01 ± 1.51	1.90 ± 0.04	8.10 ± 0.10	9.01 ± 0.06	n.d.	0.20 ± 0.01	n.d.	66.22 ± 1.72	1.7	0.1	0.4	0.4	n.d.	-	n.d	2.6
Cold pressure extraction																		
Presscake			80.10 ± 1.47	3.01 ± 0.03	14.00 ± 0.19	14.71 ± 0.23	0.19 ± 0.04	0.31 ± 0.01	0.12 ± 0.01	112.44 ± 1.98	2.9	0.1	0.6	0.7	-	-	-	4.3

**Table 4:** *Lunaria annua* seed oil fatty acid (FA) composition measured on a methyl ester basis. FA percentage is reported as the average value of two independent analyses.

Fatty Acid (FA)		Content (% on Total FAs)
Semisystematic Name (Trivial Name)		
Hexadecanoic acid (palmitic acid)	C16:0	1.3 ± 0.1 ^1,2^
Octadecanoic acid (stearic acid)	C18:0	0.1 ± 0.0
Total saturated fatty acids (SFAs)		1.4 ± 0.1
(Z)-Octadec-9-enoic acid (oleic acid)	C18:1 ω9	24.2 ± 1.1
(Z)-Icos-11-enoic acid (gondoic acid)	C20:1 ω9	0.9 ± 0.4
(Z)-Docos-13-enoic acid (erucic acid)	C22:1 ω9	44.6 ± 0.1
(Z)-tetracos-15-enoic acid (nervonic acid)	C24:1 ω9	21.8 ± 0.8
Total monounsaturated fatty acids (MUFAs)		91.5 ± 2.4
(9Z,12Z)-octadeca-9,12-dienoic acid (linoleic acid)	C18:2 ω6	6.4 ± 0.2
(9Z,12Z,15Z)-octadeca-9,12,15-trienoic acid (linolenic acid)	C18:3 ω3	1.0 ± 0.1
Total polyunsaturated fatty acids (PUFAs)		7.4 ± 0.3

^1^ duplicate; ^2^ standard error.

## Data Availability

Data are contained within the article.
